# Baseline Proteinuria and Serum Creatinine Concentration as Clinical Predictors of Complete Renal Response in Patients with Lupus Nephritis: A Single-Center Experience

**DOI:** 10.3390/ijerph192315909

**Published:** 2022-11-29

**Authors:** Andrzej Konieczny, Izabela Kasenberg, Aleksandra Mikołajczak, Piotr Donizy, Agnieszka Hałoń, Magdalena Krajewska

**Affiliations:** 1 Department of Nephrology and Transplantation Medicine, Wroclaw Medical University, Borowska 213, 50-556 Wroclaw, Poland; 2 Division of Clinical Pathology, Department of Clinical and Experimental Pathology, Wroclaw Medical University, 50-556 Wroclaw, Poland

**Keywords:** systemic lupus erythematosus, lupus nephritis, proteinuria

## Abstract

The aim of the study was to identify robust predictors of complete renal response (CRR), within 36 months, in a single-center cohort of lupus nephritis (LN) patients. Patients with biopsy-confirmed LN who underwent kidney biopsy between 1 January 2010 and 31 December 2020 were included and followed up for at least 6 months. CRR was defined as a reduction of urinary protein-to-creatinine ratio (UPCR) below 0.50 g/g. We evaluated baseline demographic, laboratory, and biopsy characteristics as potential predictors of CRR, and selected the variables further evaluated with Kaplan–Meier curves and log-rank tests. The traits with a *p*-value < 0.1 were later tested with both uni- and multivariable Cox proportional hazard models. Our sample consisted of 57 patients (84% females, median age 32 years), out of which 63.2% reached CRR within 36 months. The initial UPCR and estimated glomerular filtration rate (eGFR) were the only variables in multivariable Cox regression model, which were selected through backward elimination, with a significance threshold <0.05 (HR = 0.77, *p* = 0.01 and HR = 1.02, *p* = 0.001). Our results confirmed the role of initial UPCR and serum creatinine concentration (sCr) as predictors of CRR in LN.

## 1. Introduction

Around 30 to 60% of patients suffering from systemic lupus erythematosus (SLE) are at risk of renal involvement called lupus nephritis (LN) [1,2,3,4,5]. The clinical picture of LN ranges from an asymptomatic active urinary sediment to nephrotic range proteinuria or even a rapid progressive glomerulonephritis (RPGN) [6]. The wide symptomatology and possible discrepancy between clinical findings and histological activity make kidney biopsy a useful tool in confirming the diagnosis, which is underlined in both EULAR/ERA-EDTA and KDIGO guidelines [7,8]. The following assessment of renal tissue specimens should be performed in accordance with The International Society of Nephrology/Renal Pathology Society classification (INS/RPS). The assignment to one of six classes of LN, together with the clinical presentation, determines the subsequent treatment [7,8].

The initial goal of therapy is the normalization of estimated glomerular filtration rate (eGFR) and reduction of proteinuria, expressed by urine protein-to-creatinine ratio (UPCR) below 0.50–0.70 g/g within 1 year, defined as complete clinical response (CCR) [7]. The latter criterion is based on the results of clinical trials, which proved that proteinuria status at the 12th month is the best single early predictor of long-term kidney function [9,10]. However, not all patients achieve this target within the mentioned period. Some may require an extension of already-started therapy, but others will not reach the desired UPCR without a switch of immunosuppression regimen.

Considering the facts, establishing the probability of CCR within a longer period than 1 year seems to be crucial for making informed decisions about treatment changes. There are observational studies that have tried to meet this challenge, such as the work of Luis et al. [11]. However, the diversity of LN burden between different populations makes it reasonable to verify the significance of already-identified prognostic factors within other groups [12]. In our work, we have attempted to identify a clinical factor assessed at the time of diagnosis predicting CRR in patients with LN.

## 2. Materials and Methods

### 2.1. Study Design and Patient Selection

We conducted a retrospective single-center cohort study analyzing the medical records of 798 patients who underwent native kidney biopsy from 1 January 2010 to 31 December 2020, and identified 78 cases of LN.

**Figure 1:**
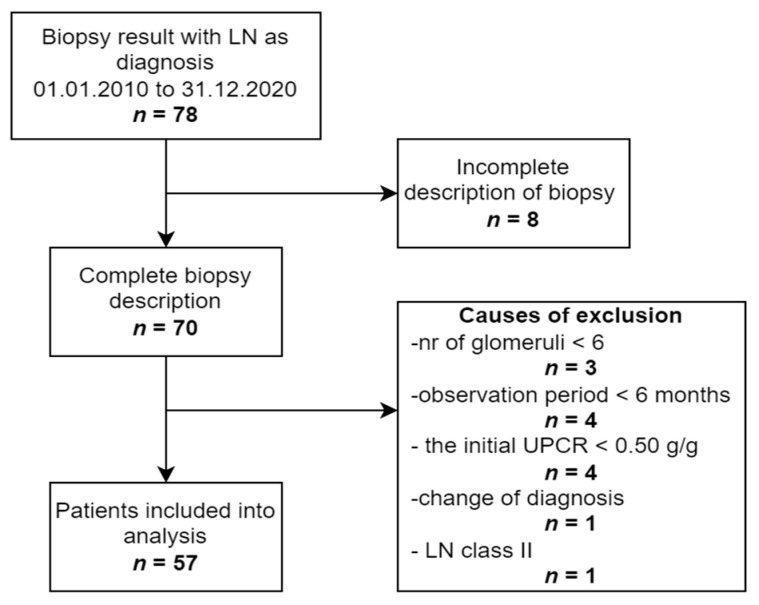
The process of patients’ selection. Abbreviations: LN, lupus nephritis; *n*, number of patients; UPCR, urine protein-to-creatinine ratio.

The following inclusion criteria were used: complete pathological description of biopsy according to the ISN/RPS classification; LN class III, IV, or V; age over 18 at the time of biopsy; confirmed clinical and laboratory diagnosis of SLE according to ACR criteria (1997). Exclusion criteria were as follows: less than 6 glomeruli in the biopsy sample; a follow-up time shorter than 6 months; a spot UPCR < 0.50 g/g at the time of biopsy. We took into consideration both patients with LN de novo, as well as with LN flare. Eventually, 57 patients were included into the analysis. The whole process of selection is summarized in Figure 1.

### 2.2. Definitions

The baseline was defined as the time of kidney biopsy. The outcome was classified as complete renal response (CRR) or no renal response (NRR). CRR was defined as UPCR under 0.50 g/g in at least two consecutive samples, whereas NRR was equal to failure to achieve this condition. The UPCR limit was set in accordance with the KDIGO 2021 guidelines for the management of LN [7]. We defined the remission focusing only on proteinuria because its reduction has already been described as the most important predictor of long-term kidney outcome [9,10]. Additionally, the eGFR from before the active disease was often unavailable, making the judgment of its normalization or improvement unfeasible.

### 2.3. Laboratory Assessment

The demographic data, laboratory values, and treatment regimens were acquired from the electronic medical records. The cut-off values were based on the local laboratory norms. Antinuclear antibodies (ANA) were measured by indirect immunofluorescence, and the titer greater than 1:100 was regarded as positive. Both C3 and C4 complement component concentrations were classified as low when their values were below 0.9 g/L and 0.1 g/L, respectively. Anti-double-stranded DNA antibody titer was measured by nucleosome-complexed enzyme-linked immunosorbent assay (anti-dsDNA-NcX ELISA). The eGFR was calculated using the MRDR equation: 186 × [serum creatinine (sCr) (mg/dl)]^−1.154^ × [age (years)]^−0.203^ × (0.762 if female).

### 2.4. Histological Findings

Biopsy results were gathered from the renal biopsy registry. The kidney samples were obtained through a percutaneous needle biopsy and later assigned to 1 of 6 LN classes according to the ISN/RPS classification by two experienced pathologists. Most cases had activity and chronicity index calculated (n = 53).

### 2.5. Statistical Analysis

Patients were divided into those who achieved CRR within 36 months from biopsy, and those not meeting this requirement qualified as NRR. The cases lost to follow-up within the observation period were treated as non-responders and their results were censored at the last visit.

To compare the characteristics of both groups, we calculated their descriptive statistics. Categorical variables were expressed as absolute numbers (frequency), and Fisher’s exact test was used to compute the statistical differences between the groups. Continuous variables were first assessed for normality using the Shapiro–Wilk test and normal probability plots. They were later presented either as mean ± SD if they were normally distributed or as median (interquartile range (IQR)) if they did not fulfill these conditions. Their comparison was performed using the unpaired t-Student test or the Mann–Whitney U test as appropriate.

We selected the potential predictors of CRR considering those already reported in the literature and having potential clinical significance [11,13,14,15]. Among the tested characteristics at baseline were: sex; age at the time of biopsy (≤25, 26–35, 36–45, and ≥46 years old); LN class (III, IV or IV/V, V); activity index (AI) (0–5, 6–11, 12–24 points); chronicity index (CI) (0–2, 3–5, 6–12 points) and its two components separately—total glomerulosclerosis (<10% vs. ≥10%) and interstitial fibrosis (<10% vs. ≥10%); eGFR (<60 vs. ≥60 mL/min/1.73 m^2^); UPCR (<2 vs. ≥2 g/g); anemia (yes vs. no); thrombocytopenia (yes vs. no); complement C3 and C4 concentration (low vs. normal). 

First, the Kaplan–Meier curve and log-rank test were estimated for each chosen predictor. The variables with a *p*-value < 0.1 in the log-rank test were included in the estimation of the univariable Cox proportional hazards regression model for predicting CRR within 36 months. The values of UPCR, eGFR, and CI were incorporated as continuous covariates, whereas other significant predictors were treated as categorical. The multivariable Cox analysis was first conducted including all predictors having statistical significance, and then using the backward elimination method (Wald-base, *p* < 0.05 as significance level). The proportional hazard assumption was checked using the statistical test based on the scaled Schoenfeld residuals. The linearity assumption was tested by plotting the martingale residuals against the continuous variables. In cases of missing data, the case was eliminated from the specific analysis. Eventually, we also calculated Spearman’s rank correlation coefficients for the covariates included in the full Cox regression model to assess the potential relationships between them. 

A graphical summary of the statistical analysis is available in Supplementary materials Figure S1.

All statistical analyses were performed by STATISTICA v. 13.3 software (TIBCO Software Inc., Palo Alto, CA, USA), and a two-tailed *p*-value of < 0.05 was considered significant if not stated otherwise.

## 3. Results

### 3.1. Patients’ Characteristics

The analysis included 57 patients after the selection, as depicted in Figure 1. All of them were of Caucasian origin, and females constituted 84% of the total sample. The age ranged between 18 and 72, with a median of 32 years. The ISN/RPS histological classes were distributed as follows: class III—12 patients (21.1%), class IV/IV + V—37 patients (64.9%), and class V—8 patients (14.0%). The membranous component was described in 32.4% (N = 12) of patients with class IV. In most instances, the induction consisted of either mycophenolate mofetil (MMF, 35.1%) or intravenous cyclophosphamide (CYC, 31.6%) or calcineurin inhibitors (CNI, 15.8%). Four patients (7.0%) obtained two medications during induction—MMF switched to CYC or MMF replaced by CNI, due to MMF intolerance. The remaining six subjects (10.5%) received only glucocorticoids. 

The clinical, laboratory, and histological characteristics of the total sample at baseline are summarized in the first column of Table 1.

### 3.2. Comparison of the Patients with CRR and NRR

Thirty-six (63.2%) out of fifty-seven patients achieved CRR within the 36-month observation period. Their median time to remission was 4 months (range: 1–30) and, in most cases (91.7%), this endpoint was met during the first year. As presented in Table 1, there were no significant differences in demographic characteristics between patients with CRR and without it. The induction regimen and concomitant drugs (hydroxychloroquine, ACEI) also appeared to be uncorrelated with achieving remission in our sample. Among the laboratory parameters, sCr, eGFR, and UPCR were significantly different between the studied groups. The patients that reached CRR initially had better kidney function. UPCR at baseline was higher in the NRR group. Both subsets of patients did not differ significantly in any serological parameter.

The kidney biopsy results of complete renal responders were characterized by lower values of CI (two vs. four in non-responders, *p* = 0.005). Out of the two examined components of CI, only the extent of interstitial fibrosis turned out to be significantly higher in NRR group, whereas total glomerulosclerosis (*p* = 0.17) did not.

### 3.3. Predictors of CRR within 36 Months

The results of the univariable Cox proportional hazard regression for predicting CRR within 36 months are presented in Table 2. 

Apart from total glomerulosclerosis and class V of LN, all the other variables showed an association with CRR. One-unit increment in eGFR was the most significant positive predictor of CRR, being considered alone (*p* = 0.0001). Male gender also indicated an association with the increased chance of remission (*p* = 0.03), but its 95% confidence interval was broad (1.10–5.38). UPCR was the only significant laboratory negative predictor (HR = 0.72, *p* = 0.002). Factors such as anemia, thrombocytopenia, and concentration of C3 or C4 were not even included in the further analysis because their *p*-values, for the log-rank test, were greater than 0.1 in Kaplan–Meier analyses. Among the biopsy findings, having LN class IV/IV + V showed the highest negative association with reaching remission (HR = 0.33, *p* = 0.005). Our results also demonstrated that CI was more helpful in predicting CRR than looking at its two analyzed components separately (*p* = 0.008 vs. *p* = 0.02 and *p* = 0.09).

Subsequently, two multivariable Cox proportional hazard models were calculated. The first one included all significant variables without interstitial fibrosis, whereas the second one was created using the backward elimination method, with a significance threshold of 0.05. The full model identified only eGFR as a significant predictor with HR = 1.02 and *p* = 0.02 (available in Supplementary materials Table S3). The backward elimination analysis, presented in Table 2, identified both UPCR and eGFR as significant covariates, with *p*-values equal to 0.01 and 0.002, respectively.

The correlation matrix for the full model’s covariates revealed moderate, but significant, correlations between eGFR and CI (Spearman’s rho = −0.55, *p* < 0.0001), as well as between eGFR and UPCR (Spearman’s rho = −0.33, *p* = 0.01), available in Supplementary materials Table S4.

## 4. Discussion

We analyzed the single-center cohort of patients with LN and described the characteristics of our sample and then determined the predictors of CRR within 36 months from the kidney biopsy. We have found that (1) the initial eGFR and UPCR were the two most robust predictors in the multivariable analyses; (2) the inclusion of CI did not improve the multivariable Cox regression model predicting remission of proteinuria in our sample; and (3) inflammatory parameters (CRP, ESR, PLR, PNR, NLR) and urine sediment did not differ between CRR and NRR groups at baseline among our patients.

Our results concerning baseline proteinuria corresponded to the observations of other researchers. The negative impact of its higher values on achieving CRR within three years from induction has already been shown by Luis et al., who demonstrated that the initial proteinuria <2 g/day was the only significant predictor of CRR in the multivariable Cox proportional hazard model, with HR = 2.32 and *p* < 0.01, compared with patients having values above this threshold [11]. Malvar et al. observed that the patients who achieved CRR at the sixth month from the beginning of the induction had significantly lower daily proteinuria at baseline in comparison to those who did not meet the remission criteria (2.2 g/d vs. 3.5 g/d, *p* = 0.018) [13]. The study by Moroni et al. presented similar results—in their cohort, the baseline 24 h proteinuria was lower in participants who responded completely to therapy within six months, compared to those who met this goal between the sixth and twelfth month (*p* < 0.01) [14]. In our descriptive statistics, higher values of UPCR characterized the NRR (4.40 g/g vs. 1.81 g/g, *p* = 0.002). In the proportional hazard Cox regression, one unit increase of proteinuria showed a negative association with attaining complete remission, both as the independent covariate (HR = 0.72, *p* = 0.002) and as an element of the multivariable analysis (HR = 0.77, *p* = 0.01). In this way, our outcomes confirmed the importance of the initial UPCR as a predictor of CRR.

The previous papers did not allow a definitive statement to be made about the importance of initial sCr and eGFR in prognosing remission. The analysis of Pirson et al. did not show any significant difference in baseline value of eGFR (*p* = 0.44) and sCr (*p* = 0.29) between patients who ever attained remission and those who never did [15]. Similar observations were reported by Malvar et al., who observed patients for six months from the first biopsy—the values of initial sCr were comparable in both complete responders and partial/non-responders [13]. By contrast, Dall‘Era et al. demonstrated in the Aspreva Lupus Management Study that eGFR <30 mL/min/1.73 m^2^ was one of the three significant predictors of renal response at the sixth month, where the responsiveness was equal to the current KDIGO definition of the partial response (OR = 0.2; CI 95% 0.1–0.4) [16]. Further observation of the same population proved that the initial eGFR ≥ 90 mL/min/1.73 m^2^ showed an independent positive association with the complete response, but only among those who already had responded to the induction (OR = 2.0, *p* = 0.04) [17]. Korbet et al. also reported similar findings in the trial comparing the standard induction scheme with the protocol with the addition of plasmapheresis—patients with complete remission turned out to have a lower sCr at the beginning of the study, independently of the applied therapy [18].

In our analysis, eGFR showed to be the best predictor, with the highest correlation with the outcome. Complete responders had almost twice as high median eGFR as the patients without remission (78 vs. 40 mL/min/1.73 m^2^, *p* < 0.0001). A similar tendency was also depicted in the results of Cox model analyses. The one-unit change in eGFR was the most significant covariate in the univariable and multivariable Cox regression models. Our results could be explained by the fact that we included both patients with LN de novo and those who underwent the biopsy due to LN flare or unsatisfactory response. The second group, by definition, could be expected to have more devastating kidney tissue damage with lower eGFR and, as the study of Ioannidis et al. proved, may need a longer time to attain re-remission [19]. Their contribution to the model, together with the detected correlation between eGFR and CI, was probably responsible for assigning eGFR such a high statistical significance in the final Cox regression model. This finding underlined the need of paying attention to the initial status of patients when including sCr or eGFR and biopsy results together into one predictive model.

The CI was significantly higher among non-responders in our sample and turned out to be an important negative predictor in the univariable analysis. However, the backward elimination method did not incorporate it into the final model. The probable cause of this was the correlation with eGFR, which caused the inclusion of CI to be statistically insignificant. It is important to keep this in mind because the previous papers reported that the initial value of CI was linked to the achievement of remission. Moroni et al. identified CI as one of two significant baseline predictors of no response to therapy at 1 year (OR = 1.22, *p* = 0.018) [14]. Park et al. also described an association between CI and CRR after one year of immunosuppressant treatment in both uni- (OR = 0.706, *p* = 0.026) and multivariable logistic regression models (OR = 0.55, *p* = 0.013) [20]. The research of Helget et al. identified CI as the predictor of a lack of complete response at 1 year with the highest cross-validated AUC alone and included it as an important variable in three out of five calculated multivariable models [21].

The other analyzed variables were not helpful in prognosing CRR. The degree of hematuria did not differ between both groups of patients and, therefore, was not considered in further analysis. This observation corresponded with the results of the Euro-Lupus nephritis trial, which demonstrated that adding the change of urinary RBC status from baseline decreased the sensitivity of the predictive model [10]. The initial values of inflammatory parameters also were not associated with assignment to CRR or NRR group. It applied to both the standard (CRP, ESR) and novel parameters (PLR, PNR, NLR). What was a little surprising to us was the matter of sex. In our population, being male was connected with a higher chance of attaining remission in the univariable Cox regression model, whereas other research demonstrated that the rate of males achieving complete remission is lower or equal to females [22]. Our results could be explained by a small representation of males in the sample, and a coincidence rather than a significant observation. This presumable explanation could be supported by the wide coincidence interval of the hazard ratio.

The strength of our study is limited by a few facts. First, the retrospective character of the study related to the irregularity of check-up visits and missing patients within the 36 months of the follow-up period. Secondly, our population consisted only of Caucasians from a restricted area, which did not enable the application of our results to different ethnic groups. Thirdly, we could not consider the impact of the treatment regime because of no standardized drug doses. Fourthly, the drawback was also the impossibility of including anti-dsDNA antibodies titer into the analysis because of numerous missing data. Further prospective studies including more heterogeneous samples would be useful to confirm our results.

## 5. Conclusions

In summary, this study confirms the role of initial proteinuria in predicting the achievement of UPCR below 0.5 g/g within 36 months from the biopsy. It also draws attention to the baseline LN status of patients by creating predictive models. Including at once LN de novo and relapses or non-responsive cases of the disease may cause the omission of significant predictors when the analysis is performed without conscious checking of the correlation between the variables. The example from our study is the relationship between eGFR and CI, which we suggest should be further evaluated in future research.

## Figures and Tables

**Table 1:** Baseline characteristics at the time of renal biopsy according to the remission status within 36 months of the observation period.

Variable ^a^	All Patients (n = 57)	Complete Renal Remission (n = 36)	No Renal Remission (n = 21)	*p*-Value ^b^
Female, *n* (%)	48 (84)	28 (78)	20 (95)	0.13
Age (years)	32 (6–45)	32 (26–45)	34 (25–39)	0.73
sCr (mg/dl)	1.10 (0.86–1.53)	0.98 (0.81–1.17)	1.67 (1.16–2.02)	**0.0001**
eGFR (ml/min/1.73 m^2^)	67 (48–83)	78 (58–85)	40 (30–61)	**<0.0001**
UPCR (g/g) ^1^	2.37 (1.38–4.62)	1.81 [1.11–3.10]	4.40 (1.99–6.42)	**0.002**
Hematuria (RBC/hpf), *n* (%)				
<5	13 (22.8)	7 (19.4)	6 (28.6)	0.53
5–20	22 (38.6)	16 (36.1)	6 (42.9)	
>20	22 (38.6)	13 (44.4)	9 (28.6)	
Total protein (g/dL)	5.2 (4.6–5.9)	5.2 (4.5–6.2)	5.2 (4.8–5.7)	0.71
Albumin (g/dL)	2.9 ± 0.6	2.9 ± 0.6	2.8 ± 0.6	0.60
Total cholesterol (mmol/L) ^2^	7.0 ± 2.2	7.1 ± 2.4	6.9 ± 2.0	0.77
Triglycerides (mmol/L) ^3^	2.1 (1.6–2.7)	2.1 (1.6–2.7)	1.8 (1.2–2.7)	0.71
Hemoglobin (g/dL)	11.9 ± 1.7	12.2 ± 1.7	11.5 ± 1.7	0.14
Leukocytes (×10^9^/L)	7.95 ± 3.05	7.75 ± 3.26	8.30 ± 2.69	0.52
Lymphocytes (×10^9^/L) ^4^	1.75 ± 0.95	1.71 ± 0.83	1.82 ± 1.14	0.67
Neutrophiles (×10^9^/L) ^4^	5.52 ± 2.54	5.33 ± 2.68	5.82 ± 2.33	0.49
Platelets (×10^9^/L)	221 (181–269)	205 (164–248)	243 (210–287)	**0.03**
NLR^4^	3.03 (1.83–5.47)	2.58 (1.79–5.41)	3.50 (2.04–5.47)	0.54
PLR^4^	129.7 (90.9–253.1)	131.7 (87.9–227.4)	119.7 (105.2–334.6)	0.49
PNR^4^	41.65 (30.05–59.70)	38.73 (30.05–56.43)	46.26 (31.62–59.70)	0.57
CRP (mg/L) ^1^	1.43 (0.43–2.97)	1.61 (0.52–2.99)	1.10 (0.35–2.65)	0.87
ESR (mm/h) ^5^	29 (21–49)	26 (22–35)	36 (19–57)	0.12
ANA positivity, *n* (%)	56 (98.2)	36 (100.0)	20 (95.2)	1.00
Anti-dsDNA Ab titer (IU/mL) ^6^	285.7 (144.9–512.1)	351.1 (178.2–560.1)	202.8 (127.2–261.6)	0.16
Low C3, *n* (%) ^3^	30 (66.7)	21 (70.0)	9 (60.0)	0.52
Low C4, *n* (%) ^7^	19 (43.2)	16 (51.6)	3 (23.1)	0.10
MMF, *n* (%)	24 (42.1)	13 (36.1)	11 (52.4)	0.27
CYC, *n* (%)	20 (35.1)	12 (33.3)	8 (38.1)	0.78
CNI, *n* (%)	11 (19.3)	9 (25.0)	2 (9.5)	0.19
HCQ, *n* (%)	26 (45.6)	17 (47.2)	9 (42.9)	0.79
ACEI, *n* (%)	25 (43.9)	16 (44.4)	9 (42.9)	1.00
ISN/RPS class, *n* (%)				
III	12 (21.1)	10 (27.8)	2 (9.5)	0.29
IV/IV + V	37 (64.9)	21 (58.3)	16 (76.2)	
V	8 (14.0)	5 (13.9)	3 (14.3)	
Activity index ^2^	8 (6–12)	8 (6–12)	10 (6–14)	0.24
Chronicity index ^2^	3 (1–4)	2 (1–4)	4 (3–6)	0.005
Proportion of glomeruli (%)				
with cellular/fibrocellular crescents ^1^	0 (0–7)	0 (0–3]	0 (0–13)	0.15
with fibroid necrosis ^1^	0 (0–17)	0 (0–14)	7 (0–18)	0.46
Total glomerulosclerosis, *n* (%)				
<10%	24 (42.1)	18 (50.0)	6 (28.6)	
≥10%	33 (57.9)	18 (50.0)	15 (71.4)	0.17
Interstitial fibrosis, *n* (%)				
<10%	40 (70.1)	30 (83.8)	10 (47.6)	**0.008**
10–25%	16 (28.1)	6 (16.7)	10 (47.6)	
25–50%	1 (1.8)	0 (0.0)	1 (4.8)	
>50%	0 (0.0)	0 (0.0)	0 (0.0)	

^a^ Data are expressed as median (interquartile range), mean ± standard deviation, or number (proportion). ^b^ *p*-values were bolded for variables with statistically significant difference between the groups. Number of missing data: ^1^ n = 1, ^2^ n = 4, ^3^ n = 11, ^4^ n = 2, ^5^ n = 10, ^6^ n = 28, ^7^ n = 12. Abbreviations: ACEI, angiotensin-converting-enzyme inhibitors; ANA, antinuclear antibodies, anti-dsDNA Ab, anti-double stranded DNA antibody; C3, complement component 3; C4, complement component 4; CNI, calcineurin inhibitors, CRP, C-reactive protein; CYC, cyclophosphamide; eGFR, estimated glomerular filtration rate; ESR, erythrocyte sedimentary rate; HCQ, hydroxychloroquine; ISN/RPS, The International Society of Nephrology/Renal Pathology Society; MMF, mycophenolate mofetil; N, number; NLR, neutrophil-to-lymphocyte ratio; PNR, platelets-to-neutrophil ratio; PLR, platelets-to-lymphocyte ratio; sCr, serum creatinine; RBC/hpf, number of red blood cells per high power-field; UPCR, urine protein/creatinine ratio.

**Table 2:** Results of uni- and multivariable Cox proportional hazard regression models for predicting complete renal response within 36 months.

	Univariable			Multivariable		
Variable	HR	95% CI	*p*-Value	HR	95% CI	*p*-Value
Male, vs. female sex	2.44	1.10–5.38	0.03	—	—	—
eGFR mL/min/1.73 m^2^	1.02	1.01–1.04	0.0001	1.02	1.01−1.03	0.002
UPCR g/g	0.72	0.58–0.89	0.002	0.77	0.62−0.95	0.01
LN class				—	—	—
III (ref.)	(1.00)					
IV or IV + V	0.33	0.15–0.72	0.005			
V	0.35	0.12–1.04	0.06			
Chronicity index	0.79	0.67–0.94	0.008	—	—	—
Interstitial fibrosis ≥10%, vs. <10%	0.36	0.15–0.87	0.02	—	—	—
Total glomerulosclerosis ≥10%, vs. <10%	0.56	0.29–1.07	0.08	—	—	—

HR for covariates was calculated for one unit increase of the variable. Abbreviations: 95% CI, 95% confidence interval; eGFR, estimated glomerular filtration rate; HR, hazard ratio; LN, lupus nephritis; UPCR, urine protein-to-creatinine ratio.

## Data Availability

The data presented in this study are available in Supplementary Materials.

## Supplementary Materials

The following supporting information can be downloaded at: https://www.mdpi.com/article/10.3390/ijerph192315909/s1, Figure S1: Graphical summary of the performed statistical analyses; Table S1: Comparison of all calculated multivariable Cox regression models; Table S2: Correlation between covariates in the full multivariable Cox regression model.
