# Supplementary material for: Baseline Proteinuria and Serum Creatinine Concentration as Clinical Predictors of Complete Renal Response in Patients with Lupus Nephritis: A Single-Center Experience
Source: Int J Environ Res Public Health. 2022 Nov 29;19(23):15909. doi: 10.3390/ijerph192315909 (PMC9737901; doi:10.3390/ijerph192315909)
Supplement: Supplementary file 1 [file ijerph-19-15909-s001.zip › ijerph-2007143-supplementary.pdf]

SUPPLEMENTARY MATERIALS

Figure S1. Graphical summary of the performed statistical analyses

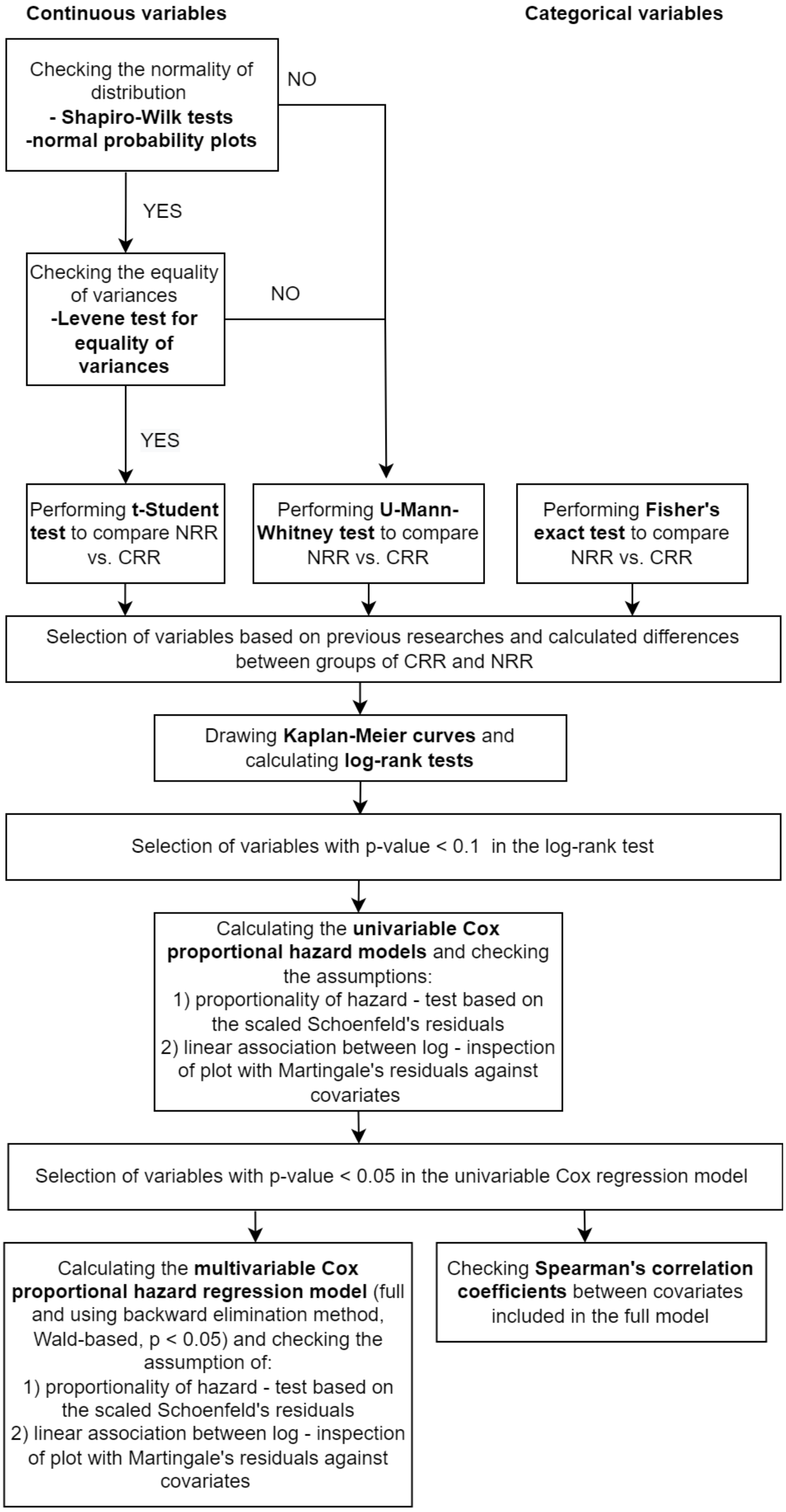

Table S1. Comparison of all calculated multivariable Cox regression models

	The full multivariable model (n=52)			*The multivariable model unadjusted for sex (n = 52)*			*The final model obtained through backward elimination method (n = 56)**		
**Variable**	**HR**	**95% CI**	***p*-v.**	**HR**	**95% CI**	***p*-v.**	**HR**	**95% CI**	***p*-v.**
Male vs. female (ref.)	2.48	0.91 to 6.73	0.08	—	—	—	—	—	—
eGFR ml/min per 1.73m^2	**1.02**	**1.00 to 1.03**	**0.02**	1.01	1.00 to 1.03	0.06	**1.02**	**1.01 to 1.03**	**0.002**
UPCR g/g	0.80	0.62 to 1.03	0.08	**0.77**	**0.60 to 0.99**	**0.04**	**0.77**	**0.62 to 0.95**	**0.01**
LN class									
III (ref.)	1.00			1.00					
IV or IV+V	0.55	0.23 to 1.31	0.18	0.62	0.26 to 1.48	0.28	—	—	—
V	0.79	0.15 to 4.07	0.77	0.78	0.15 to 4.03	0.77			
Chronicity index	0.92	0.74 to 1.14	0.45	0.89	0.72 to 1.10	0.27	—	—	—

Abbreviations: 95% CI, 95% confidence interval, HR, hazard risk; p-v., p-value

*The variables were first identified using backward elimination method with p-value < 0.05 as threshold and starting from the full multivariable model. Subsequently, the selected covariates were used to calculate the final model. In this way, we could increase the number of cases included into the final analysis because we could include the patients without calculated chronicity index.

Table S2. Correlations between covariates in the full multivariable Cox regression model

Pair of variables	*n*	Spearman's rho	p-value
Chronicity index and eGFR	**53**	**-0.55**	**< 0.0001**
Chronicity index and UPCR	52	0.18	0.20
eGFR and UPCR	**56**	**-0.33**	**0.01**
